# Wellbeing and Retirement in Europe: A Systematic Review with Meta-Analysis

**DOI:** 10.3390/healthcare13020100

**Published:** 2025-01-07

**Authors:** Andreia P. Teques, Joana Carreiro, Daniel Duarte, Pedro Teques

**Affiliations:** 1 N2i, Polytechnic Institute of Maia, 4475-690 Maia, Portugal; apereirateques@ipmaia.pt (A.P.T.); dfduarte@ipmaia.pt (D.D.); 2 Department of Social Sciences and Behavior, University of Maia, 4475-690 Maia, Portugal; jcarreiro@umaia.pt; 3 Research Center in Sports Sciences, Health Sciences and Human Development, 5001-801 Vila Real, Portugal

**Keywords:** Europe, health consequences, meta-analysis, retirement, systematic review, wellbeing

## Abstract

**Background/Objectives**: Given the observed increase in life expectancy within Europe, it is anticipated that there will be a growing number of retirees and a lengthening of the retirement phase for individuals. This has brought attention to the examination of how the disengagement from professional endeavors influences overall wellbeing. The objective of this investigation was to conduct a comprehensive literature review spanning the period from 1998 to 2024, focusing on the intersection of retirement and wellbeing. **Methods**: This review (PROSPERO reference: CRD42024621454) was based on scientific articles available in PsycInfo, PubMed, Scopus, SPORTDiscus, and Web of Science, resulting in the inclusion of 32 articles in the systematic review, of which 12 were eligible for meta-analysis. The quality of evidence was evaluated based on the Quality Assessment Tool for Observational Cohort and Cross-Sectional Studies checklist. **Results:** The meta-analysis results revealed that the observed effect size was d = 0.383, considered moderate. This value was statistically significant (Z = 3.197; *p* = 0.001) with a 95% CI [0.148, 0.617]. The publications were subjected to qualitative analysis, taking into account study type and thematic content. The discernible outcomes were categorized as follows: (1) expectations regarding retirement, (2) preparation for retirement, (3) family relations and grandparenting, (4) quality of life and satisfaction with retirement, and (5) health consequences of retirement. **Conclusions**: The prevalent approach predominantly comprised quantitative investigations, with a particular focus on exploring the nexus between retirement and health implications, particularly in the context of European participants. This emphasis on health consequences provides a deeper comprehension of this association.

## 1. Introduction

The extension of life expectancy and the aging of the population are topics that have been gaining a significant attention. European countries have been undertaking important studies and investments related to this topic, considering that the increase in the longevity of the population has the immediate consequence of an increasing number of retired people. According to Eurostat [1], Europe expects the median age in the EU to increase by 4.5 years, reaching 48.2 years between 2019 and 2050. This change in median age is likely to have significant implications for retirees’ wellbeing, potentially affecting access to healthcare, financial sustainability of pension systems, and the availability of social support networks [2].

According to projections, the number of older people already surpasses the number of children and teenagers, and there will be close to half million centenaries in 2050. The future age pyramids of the population will see an excessive reduction in the number of citizens at their base, where there will be a narrowing, and an increase in the participation of later ages. This demographic shift is expected to influence retirees’ wellbeing, as it may strain social support systems and reduce intergenerational care availability, potentially affecting the quality and accessibility of necessary services [3].

Also, according to the Eurostat projection, Europe will continue to increase years in the population average life. In 2019, the number of women aged 85 and over was more than double that of men in the same age group. In this sense, while life expectancy increases, more pensions are granted. In 2018, expenditure on pensions exceeded EUR 1.712 billion across European countries, representing 12.7% of total economic output. An extension of around 20 years in the time that an individual remains in the condition of retirement has been observed, representing an extensive period of citizen life. Thus, this extended period offers an opportunity to explore its impact on retirees’ wellbeing, including potential challenges related to mental health, financial needs, and social engagement [4].

As retirees navigate this phase, it marks a transition from a cycle in which there is a link with work life to another guided by unemployment and leisure [5,6]. It gives the individual the real possibility of an experience of non-work, and such an experience can be perceived negatively or positively. People can feel this moment in a painful way, permeated by feelings of insecurity and doubts regarding the emptiness caused by the lack of activity, work, as well as the loss of bonds arising from the work environment. On the one hand, the breaking of the productivity cycle with work, the awareness of no longer belonging to that professional group, and the association of this phase with aging characterize ambiguous aspects in this retirement transition. On the other hand, in a positive way, the person perceives that the moment of retirement is the earned right to rest, to enjoy free time, to choose what, where, and how to carry out activities with family and friends. This phase can involve the discovery of new interests and an adjustment to a slower pace of life, with a focus on quality of life, unburdened by the rules and routines of the work environment [7].

In this context, Wang and Schultz’s [8] psychological perspective on the changing nature of retirement provides a deeper understanding of this transition. They argue that retirement is not a single event, but rather a complex, multi-phase process that involves significant psychosocial adjustments. The retirement process is conceived as a longitudinal progression with potential impacts on psychosocial factors, such as life satisfaction, lifelong activities, and physical and mental health. This framework suggests that the nature of the work environment, expectations about retirement, family factors, socioeconomic factors, and individual attitudes all play a crucial role in shaping the psychological experience of this phase [9]. Retirees who anticipate a positive experience may adjust more easily, seeing retirement as an opportunity for personal growth, whereas those who are unprepared or who view retirement negatively may struggle with poor mental health.

Throughout one’s professional career, the quality of the work environment significantly influences the emotions associated with retirement, whether positively or negatively [10]. Considering this viewpoint, the evident increase in life expectancy in Europe, and the importance of the relationship between retirement and wellbeing, this study sought to systematically investigate the current literature. A meta-analytic approach was employed, encompassing European-focused research conducted between 1998 and 2024. The main objectives of this investigation are (1) to meta-analyze the relationship between retirement and the wellbeing of the older people; (2) to identify any gender differences during retirement; (3) to analyze how socioeconomic variables (e.g., level of education, employment status) influence the experiences of the older people during retirement; and, lastly, (4) to map research trends about wellbeing in retirement and the future challenges for this growing population in Europe.

## 2. Materials and Methods

This comprehensive evaluation adhered to the guidelines outlined in the Preferred Reporting Items for Systematic Review and Meta-analyses (PRISMA) statement, as suggested by Moher et al. [11]. The review protocol is registered in PROSPERO (reference: CRD42024621454).

### 2.1. Search Strategy and Eligibility Criteria

Acknowledged scientific databases in the field of research were chosen: PsycArticles, PsicBooks, PsycInfo, PubMed, Scopus, SPORTDiscus, and Web of Science. These databases were chosen because they are considered the most comprehensive among those available and because they aggregate consistent sources of scientific data. Regarding the posting period, the range from 1998 to 2024 was considered. The exploration approach encompassed the terms put forth in the Medical Subject Headings (MeSH) as part of the search strategy and their correlates: “retirement” and “wellbeing or well-being or well being” and “european union or eu or europe”.

Only empirical articles involving qualitative, quantitative, or mixed data collection were included in the search. All search strategies were developed in July 2024. In the initial phase of the search, the entries that fulfilled the subsequent conditions were considered: (1) comprised pertinent information regarding retirement and wellbeing; (2) study participants were only European citizens; (3) included participants aged 50 years or older to explore expectations regarding retirement; and (4) were written in the Croatian, English, French, Italian, Portuguese, Serbian, Spanish, and Swedish languages. Articles were not taken into account if they fulfilled the following criteria: (1) included participants of non-EU countries, and (2) did not contain any relevant data on retirement and wellbeing.

### 2.2. Data Analysis

The titles and abstracts of all articles pinpointed through the search methodology were separately examined by two authors (APT, PT) involved in this study. In the subsequent stage, the reviewers individually appraised the complete articles and determined their inclusions based on predetermined qualifying conditions. Any differences in opinions between the reviewers were resolved through mutual agreement.

The selected studies underwent an assessment of their methodological biases using the Quality Assessment Tool for Observational Cohort and Cross-Sectional Studies checklist [12]. This checklist comprises 14 questions aimed at appraising the potential bias and methodological rigor of the studies. Each question was addressed with responses like “yes”, “no”, “not applicable”, or “not reported”. For cross-sectional studies, questions 6, 7, 8, 10, 12, and 13 were rated as N/A. The studies were then classified into categories: good, fair, or poor. The evaluation of article quality was independently conducted by two researchers. Instances of uncertainty were jointly deliberated by the researchers, and in cases of discrepancy, the viewpoint of a third researcher was incorporated.

The articles were deemed suitable for the meta-analysis if their respective studies reported the same statistical effects and considered wellbeing assessed by the same psychometric scale. These criteria were defined so that the respective effects could be interpreted appropriately. Effect sizes were extracted, and their variances were computed to apply inverse-variance weighting. Heterogeneity was assessed using Cochran’s Q and I² statistics. Fixed-effects and random-effects models were employed, depending on heterogeneity levels using IBM SPSS 29.

## 3. Results

**Figure 1:**
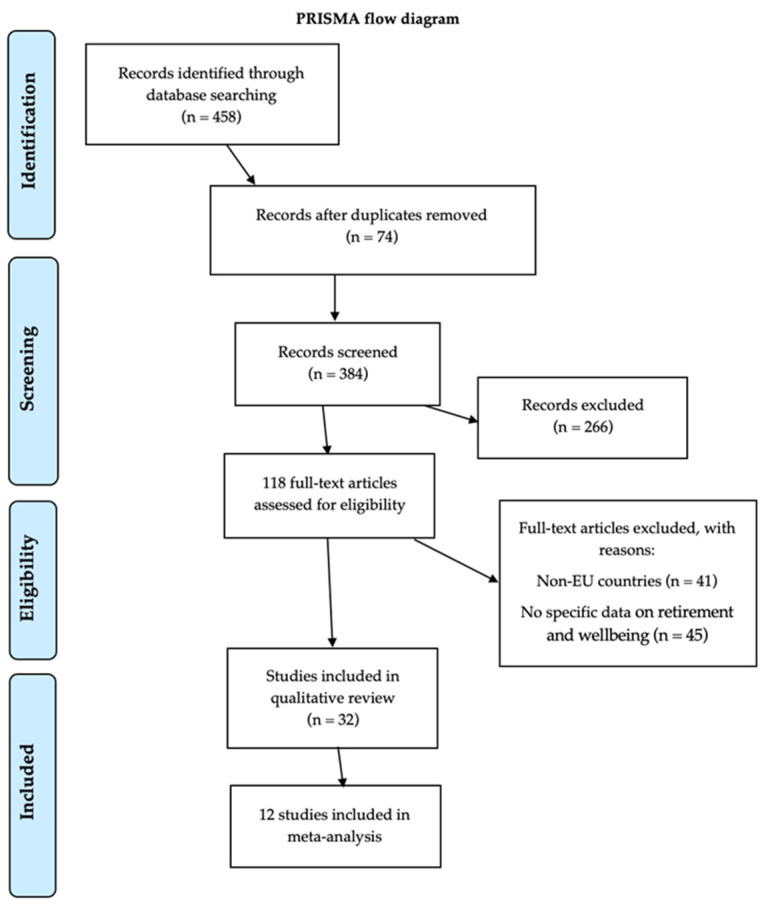
PRISMA flow diagram of the studies included in the analysis.

In the initial analysis phase, a total of 458 publications were identified. These findings were subsequently imported into EndNoteTM X8 software, where duplicate records (74 references) were systematically removed, either through automated processes or manual review. The remaining pool of articles (384) underwent scrutiny based on their titles and abstracts. After this initial screening, a careful selection was made, retaining those closely aligned with the research objectives, leading to the elimination of 266 articles. Among the remaining 118 articles, a comprehensive evaluation was conducted, resulting in the exclusion of 86 articles that did not meet the predefined inclusion criteria. Following this thorough review process, a total of 32 articles were identified for detailed examination and analysis (Figure 1). The data extracted were organized taking into consideration the publication identification, country, type of design, age group, sample characteristics, and variables involved.

### 3.1. Risk of Bias

The studies mostly showed good quality levels. In general, although they included large samples, almost all studies fundamentally lacked sample size justification, power description, or variance and effect estimates, except for Weziak-Bialowolska et al. [13], which received greater recognition. Additionally, nearly half of the studies did not provide data on key potential confounding variables or statistically adjust for their impact on the relationship between exposure and outcome. The risk of bias and quality assessment of the included studies are shown in Table 1.

### 3.2. Meta-Analysis

**Figure 2:**
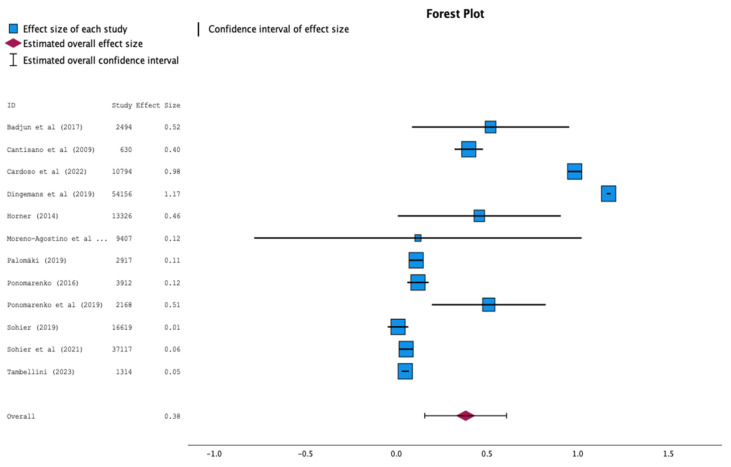
Forest plot [17,19,20,23,26,28,33,35,36,39,40,42].

Associations between wellbeing and retirement revealed effect sizes ranging from 0.01 to 1.17, indicating significant variability across studies. Most effects are small to moderate, with studies like Dingemans et al. (1.17) and Cardoso et al. (0.98) showing the largest effect sizes. The overall estimated effect is 0.38, suggesting a small to moderate positive impact on the analyzed phenomenon. In contrast, smaller studies, such as Tambellini and Cantisano et al., have less influence. The precision of the results also varies, with narrower confidence intervals in robust studies (e.g., Cardoso et al.) and wider intervals in others (e.g., Moreno-Agostino et al.), indicating uncertainties. Overall, the results indicate a consistent yet moderate effect that may be influenced by the observed variability (Figure 2).

### 3.3. Study Description and Qualitative Analysis

Table 1 shows a synthesis of all 32 selected studies. Most studies have a cross-sectional design. The participants involved were approximately 300,533 men and 257,221 women, totaling around 557,754 participants aged 50 and over. The studies cover data from a wide range of European countries. The total number of different countries is 39, reflecting the diverse European scope of the studies on retirement and wellbeing. Studies variables include wellbeing with other correlates, such as depressive symptoms, social activities, physical health, life satisfaction, and quality of life.

These investigations, predominantly centered around data from the Survey of Health, Ageing and Retirement in Europe (SHARE), were systematically grouped into five primary themes. Following the longitudinal progression of the retirement process [8], this arrangement led to the subsequent categorization: (1) expectations regarding retirement, (2) preparation for retirement, (3) family relations and grandparenting, (4) quality of life and satisfaction with retirement, and (5) health consequences of retirement. The specifics pertaining to each of these analytical domains will be expanded upon in the subsequent sections. The focal studies under consideration primarily emanate from the Survey of Health, Ageing and Retirement in Europe (SHARE), which encompasses intricate microdata covering aspects like physical and mental wellbeing, socioeconomic standing, as well as social and familial networks of the older population across Europe. Commencing in 2004, this survey has released nine waves of data, involving participants from 28 European nations plus Israel. This comprehensive dataset empowers researchers to enhance their comprehension of various facets pertaining to this stage of life. Table 2 shows the characteristics of the included studies.

#### 3.3.1. Expectations Regarding Retirement

Feelings towards retirement are characterized by a sense of hope and anticipation, with many expressing interest in continuing to work after retirement. The greatest concern raised is the potential decline in physical wellbeing [44]. Experiences of gains and losses shape the lives of participants approaching retirement. On one hand, retirement may offer an opportunity to supplement income, expand social bonds and experience, and pursue personal work projects. On the other hand, it can evoke feelings of insecurity, driven by financial instability and the loss of social roles [34]. Research also highlights the pursuit of greater freedom, improvements in family life, and enhanced quality of life, all of which have the potential to reshape personal identity [40].

Substandard working conditions are notably linked to an increased likelihood of early retirement. Siegrist et al. [38] emphasize the consistent correlation between unfavorable psychosocial work environments and the intention to retire early among senior employees across all European nations.

Likewise, Olivera and Ponomarenko [32] establish a connection between pension uncertainty and personal expectations regarding potential governmental reductions in pension benefits or an elevation of the official retirement age. This phenomenon particularly affects individuals who are farther from their retirement age, have lower income, perceive their long-term survival prospects as limited, exhibit heightened cognitive abilities, and lack expectations of private pension support.

Early retirement also tends to be more appealing to individuals employed in the private sector, those with lower levels of education, poor health, or reduced quality of life. Reforming the pension system to discourage early retirement is insufficient on its own. Bađun and Smolić [17] found that a significant number of Croatian workers would be willing to extend their careers if provided with better job opportunities, improved access to education, and enhanced healthcare. These areas appear to require substantial policy improvements.

More recently, it has been revealed that a worker’s job circumstances and partner’s retirement status are key factors influencing the decision to retire. Individuals who express a desire to retire often hold positions that impose significant cognitive and physical demands. They also report lower levels of recognition from supervisors or colleagues and fewer opportunities for career advancement. The tendency to retire is amplified when an older worker’s partner has already retired. Notably, in regions with the lowest rates of retirement inclination, employees experience more favorable working conditions and exhibit a greater ability to manage their financial obligations [41].

#### 3.3.2. Preparation for Retirement

The theory of the benefits of bridge employment was developed in response to the negative perception of retirement, which is often seen as a period of loss of previous social roles and resources [46]. This framework focuses on the transition that allows for individuals to continue their previous roles but with altered working conditions, respecting the vital needs of older people, such as autonomy, pleasure, and control. The quantity and quality of bridge employment are emphasized, as these variables are predictors of job satisfaction, life satisfaction, and quality of life in retirement [19]. In socioeconomically poorer countries, continuing to work after retirement is common, and the extra income is a significant factor in life satisfaction, particularly for retirees with small pensions and without a partner [23].

Sohier [39] explored how workers’ perceived freedom of choice in extending their careers (voluntary or forced) affects their wellbeing. The results indicated that individuals in the “forced retirement” group experienced significantly lower levels of wellbeing when they continued working. After retirement, this group reported higher life satisfaction, but the residual effects of involuntary employment persisted, leading to lower overall life satisfaction compared to those who retired voluntarily. Similarly, recent work by Sohier et al. [40] did not find significant differences in overall wellbeing between partially and fully retired individuals, those retiring before or after the standard retirement age, or those retiring concurrently with or separately from their partners. Nevertheless, for certain older workers, especially those engaged in low-quality jobs, retirement can offer relief from their current employment situation.

The association between early retirement and life satisfaction was explored by Ponomarenko [35]. Findings showed a strong negative association between early labor market exit and wellbeing in Germany, Austria, Sweden, and Denmark, despite policies promoting old-age employment. In southern European welfare states, early retirement is more frequent, with Italian women showing positive wellbeing, a contra-intuitive finding. However, more recently, Tambellini [42] evidenced certain paths, marked by periods of part-time work or interruptions, show a steady rise in life satisfaction as women transition from work (or unemployment) to retirement. Conversely, for other patterns, like full-time employment, retirement appears to have little impact on women’s personal wellbeing.

#### 3.3.3. Family Relations and Grandparenting

The wellbeing of older individuals is closely associated with their marital status. Adena et al. [15] explored the variations in wellbeing and its trajectory following widowhood for older women, comparing those who were widowed with those who remained partnered. The authors confirmed a significant decline in mental health and life satisfaction following the loss of a partner, with a gradual partial recovery over a span of five years. Despite pre-widowhood controls, such as comprehensive assessments of family ties and social networks, these factors provided limited explanatory power for the observed decline in wellbeing.

Hank and Vagner [25] examined the relationships between parenthood, marital status, and various dimensions of wellbeing in older adults, focusing on economic conditions, psychological health, and social connections. Their findings suggest that childless individuals generally do not experience lower wellbeing than parents across these dimensions. While marriage appears to provide some “protective effect”, simply having a partner does not inherently enhance psychological wellbeing. Instead, only those satisfied with the reciprocity in their relationship exhibit fewer depressive symptoms compared to their unmarried peers.

Djundeva et al. [24] identified four distinct patterns of social networks among older adults living alone. In eastern and southern Europe, “restricted” and “child-based” networks are more common, whereas “friend-oriented” networks prevail in western and northern Europe. Across countries, older adults with “restricted” networks tend to report lower wellbeing, while those with “diverse” networks demonstrate even higher wellbeing than those living with others. The study underscores the importance of differentiating older adults living alone, as the majority (around two-thirds) are not vulnerable and often experience levels of wellbeing comparable to or higher than those cohabiting with others. While country-level factors shape opportunities for developing strong social networks, individual resources and social connections play a more significant role in determining subjective wellbeing than broader societal factors.

Litwin and Levinsky [27] found that personality traits account for a greater share of the variability in wellbeing outcomes compared to social network characteristics. However, their analysis revealed that social network attributes, particularly network size and the average emotional closeness of relationships, mitigate the adverse effects of maladaptive personality traits on subjective wellbeing in later life. This highlights the critical role of social network characteristics in buffering the negative impact of certain personality traits on key indicators of wellbeing.

Research suggests that becoming a grandparent positively influences wellbeing, though evidence on the causal nature of this relationship remains limited. Danielsbacka et al. [22] highlight that grandparenthood generally enhances wellbeing, but evidence of a causal link is scarce. Their findings indicate that grandparents who provide childcare support to their adult children experience improved health and wellbeing. However, these associations are primarily attributed to differences between individuals rather than changes within the same individual over time. The only robust within-person finding was a reduction in daily living limitations among grandparents who provided childcare. This suggests a limited causal connection between grandchild care and wellbeing, with the impact likely confined to physical health rather than cognitive benefits.

Similarly, Arpino and Gómez-Léon [16] found that caring for grandchildren lowers depression risk for grandmothers but not grandfathers. However, grandparents providing intensive care to co-resident grandchildren face a higher risk of depression. For grandmothers, the protective effect of grandchild care diminishes if they also provide care for others, such as sick or disabled individuals, leading to increased depression risks. Thus, combining grandchild care with other intensive caregiving responsibilities may negate the positive effects on their wellbeing.

Bauer et al. [18] studied whether, why, and how parenthood influences wellbeing in older age. Findings revealed that fathers exhibit more favorable health trajectories and are less negatively impacted by health limitations compared to childless men. These results suggest that children may serve as a source of social control, contributing to long-term health benefits and providing a coping mechanism for fathers in the face of health challenges, whereas similar effects are not observed for mothers. Beyond family support, Xia et al. [45] emphasize the significance of balanced support exchanges between older adults and nonrelatives. This balance impacts depressive symptoms, life satisfaction, and overall subjective wellbeing.

#### 3.3.4. Quality of Life and Satisfaction with Retirement

Retirement is a life phase that cannot be universally classified as either beneficial or harmful. It is shaped by a range of factors and life experiences, both positive and negative, which influence voluntary or forced retirement decisions. Additionally, continuing to work after retirement can have mixed effects on life satisfaction, depending on individual circumstances [23].

Retirement is generally associated with a slight decline in subjective economic wellbeing [33]. However, a more detailed analysis reveals that its effects vary according to an individual’s pre-retirement employment status. For those transitioning from employment, retirement often results in a noticeable reduction in perceived income adequacy. In contrast, retirement tends to have a more positive impact on individuals transitioning from unemployment and, to a lesser extent, those shifting from other statuses.

Interestingly, for individuals moving from unemployment to retirement, there is an increase in life satisfaction, reflecting a “catching-up” effect relative to employed individuals entering retirement. For those who were previously inactive in the labor market, retirement does not significantly alter their wellbeing. Given the recovery in wellbeing observed among unemployed individuals’ post-retirement, it is essential to support the currently unemployed population to mitigate the adverse effects of economic hardship and reduced wellbeing [36].

Horner [26] suggests that the initial phase of retirement is often experienced with satisfaction. However, this satisfaction tends to decrease after a few years. Early retirement or retirement at the formal age of 65 appears to be a neutral long-term variable in terms of subjective wellbeing. Wahrendorf and Siegrist [43] highlight that engaging in and sustaining productive activities, such as volunteering or caregiving, significantly enhance the wellbeing of older adults.

From a practical perspective, Moreno-Agostino et al. [28] emphasize the importance of promoting experiential wellbeing among retirees worldwide. They recommend encouraging retirees to restructure their time in ways that enhance daily emotional experiences. Policies that support flexible time management for older adults—such as reducing working hours and time spent on work-related activities—could further contribute to their overall wellbeing.

Rojo-Perez et al. [37] classify older adults into distinct active aging profiles, drawing on four key pillars to explore how these profiles relate to personal and contextual factors, as well as wellbeing and quality of life in later years. Their analysis identifies five profiles: moderate activity, quasi-dependents, active aging with limiting conditions, diverse and balanced activity, and excellent active aging conditions. The first three profiles are associated with older adults who tend to have a higher average age, lower education levels, and are more likely to be retired or homemakers. These individuals experience moderate levels of loneliness, social satisfaction, and quality of life. They typically live in small households or alone but maintain larger family networks. In contrast, the latter two profiles are characterized by more favorable personal and contextual conditions, as well as higher levels of wellbeing and quality of life.

#### 3.3.5. Health Consequences of Retirement

Europe has one of the oldest populations in the world, yet the study of retirement’s connection to health has only recently gained attention. At present, much of the research focuses primarily on disease, disability, and mortality. However, these health outcomes do not fully capture the concept of health, which extends beyond merely the absence of disease.

Okely et al. [31] examined whether individualism influenced the relationship between wellbeing and self-rated health, as well as the link between wellbeing and mortality risk over time. Their findings revealed a significant interaction, suggesting that the association between wellbeing and self-rated health, as well as the risk of cardiovascular-related mortality, was stronger in countries with higher levels of individualism. However, this interaction did not hold when predicting all-cause mortality. These findings align with other studies that have shown a relationship between higher wellbeing and lower incidences of arthritis [24], as well as between higher wellbeing and a reduced risk of chronic lung disease [30].

Regarding mental health, Ploubidis and Grundy [34] explored how the country of residence affects depressive symptoms and wellbeing among older adults. After adjusting for demographic factors and chronic illnesses, they found that Spain had the highest levels of depression, while Denmark ranked highest in wellbeing. Better mental health was associated with higher education levels and being married. Notable differences in mental health outcomes across European countries were observed, with Scandinavian countries, the Netherlands, and Austria reporting the most favorable results (low depression, high wellbeing). Germany and France followed, while Spain, Italy, and Greece reported the poorest mental health outcomes.

Regardless of type 2 diabetes (T2D), Europeans with multiple chronic conditions, activity limitations, and health issues that restrict paid employment were more likely to be retired. On the other hand, individuals who were married or had higher levels of education were less likely to be retired. Notably, for Europeans in their fifties and early sixties, T2D alone did not significantly increase the likelihood of retiring before the age of 65. Those who retired early are likely to face financial constraints regarding healthcare due to lower education levels and the absence of a full old-age pension. In this group, T2D may pose an additional risk as they age, given its chronic and progressive nature, which can lead to complications and higher medical expenses [20].

Abramowska-Kmon et al. [14] examined the impact of receiving informal care and the perceived adequacy of care on subjective wellbeing among individuals aged 65 and older across various European countries. Their findings indicated that receiving regular informal care was linked to higher subjective wellbeing among older adults in northern Europe but to lower wellbeing among older men in southern Europe. Additionally, older adults who felt that the care they received was either insufficient or adequate reported lower wellbeing compared to those who did not require care. Receiving formal care was associated with reduced subjective wellbeing in northern, central, and eastern Europe. These results highlight the need for social policies to address caregiving comprehensively, aiming to improve the subjective quality of life for both caregivers and care recipients.

According to Weziak-Bialowolska et al. [44], engaging in volunteering or charity activities within the community on a weekly basis is associated with higher levels of wellbeing and even a lower likelihood of Alzheimer’s diagnosis. This positive view of active aging is supported by existing research, which indicates that physical activity plays a key role in fostering positive emotions among older adults. Cheval et al. [21] emphasized that physical activity is crucial in explaining the link between low educational attainment and negative mental health outcomes in adults aged 50 and older. They found that lower education levels were associated with reduced physical activity and faster declines over time, leading to greater increases in depressive symptoms and more significant declines in wellbeing.

## 4. Discussion

This study aimed to carry out a systematic review and meta-analysis of the literature produced in the last 20 years at European level on the relationship between retirement and wellbeing. The search of available the literature reinforced a longitudinal perspective of the retirement process [9] and may be organized in five topics: (1) expectations regarding retirement; (2) preparation for retirement; (3) family relations and grandparenting; (4) quality of life and satisfaction with retirement; and (5) health consequences of retirement. When comparing the results by topic, it was observed that the most studies were related to the consequences of retirement for health. This fact provided greater understanding of the transition process, the period immediately before and after retirement, as well as the interference of this phase in the health of citizens, at the time of life when the separation from work is about to occur or is already taking place.

The findings of the meta-analysis reveal a notable variability in the association between wellbeing and retirement. While most studies report small to moderate effects, a few, such as Dingemans et al. [23] and Cardoso et al. [20], stand out with large effect sizes. The overall estimated effect size of 0.38 suggests a small to moderate positive relationship between retirement and the analyzed phenomenon. Smaller studies, including Tambellini [19] and Cantisano et al. [42], exert less influence on the overall effect, likely due to their limited sample sizes. Variations in precision further highlight differences in methodological robustness, with narrower confidence intervals observed in studies like Cardoso et al., contrasting with wider intervals in others, such as Moreno-Agostino et al. [28], which indicate greater uncertainty. Despite the heterogeneity, the results collectively suggest a consistent moderate effect, although the variability warrants further investigation into potential moderating factors.

In general, qualitative findings pointed out that the age of the subject at the time of retirement can influence the repercussions on retirees’ mental health and suggested that remaining linked to some work activity can be beneficial to the health of older people. Researchers must consider this implication in the debate on longer working careers, where the worker’s perceived freedom of choice to continue working plays a crucial role in the wellbeing of older individuals [39].

Preparing for retirement is also a relevant aspect for the emotional stability of retired people. In this case, Merkel et al. [47] shows how important an education program for retirement can be, which can provide opportunities in this new phase, for the construction of a new life project. In addition, the role of the health practitioners is highlighted as a facilitator of this process, through professional guidance—in which aspects surrounding retirement can be worked out more comprehensively and at a higher level of quality.

Subjective wellbeing also plays a critical role in the decision to retire early. Research has demonstrated that individuals experiencing high levels of psychological distress, such as stress, anxiety, and depression, may retire earlier than those who report better mental health. For example, according to a study by Lahdenperä et al. [48], psychological distress is a significant predictor of early retirement, with individuals suffering from mental health issues like depression often opting to leave the workforce prematurely. The stress of dealing with chronic psychological issues can lead to a loss of motivation, poor job satisfaction, and an overall decline in wellbeing, making early retirement an appealing option. Burnout syndrome specifically has been found to increase the likelihood of early retirement, especially in high-stress jobs [49].

Another relevant topic is the quality of life and satisfaction with retirement. Researchers showed that better quality of life after retirement is associated with satisfaction with this condition [28,40]. Due to the time available due to the absence of work, retirement can allow for the practice of regular physical exercise and the expansion of space for pleasure, enabling the person to excel in interpersonal relationships and wellbeing. Thus, the retirement transition can potentially promote positive health behaviors through programs specifically designed for older adults, many of whom face physical and health constraints [37].

Also, maintaining a socially active routine, such as taking care of grandchildren, or having tasks that promote the feeling of usefulness, increase the perception of wellbeing, always with the premise of effective time management, by allowing for self-care at the same time, e.g., [24]. This could be particularly important for retirees who experience a sense of loss or a shift in identity during the “empty nest” period. The loss of daily caregiving responsibilities can lead to feelings of emptiness or a lack of purpose, particularly for those whose identity has been closely tied to their parenting roles [50]. In order to ensure that this stage of life is experienced with a sense of quality wellbeing, researchers also cannot neglect the major concerns of this age, such as health and economic limitations, emphasizing specialized technical support that allows for comprehensive support to the retired person [32].

Finally, it is important to understand that welfare systems and pension policies can play an important role in the wellbeing of European populations. European countries exhibit significant variations in their welfare systems, which may affect retirees’ financial security and, consequently, their wellbeing. For example, Nordic countries (e.g., Denmark, Sweden, Norway) have welfare systems that include universal healthcare, pension schemes, and social support networks. These countries provide retirees with financial stability, reducing concerns over healthcare costs and living expenses [51]. These systems allow for retirees to enjoy a quality of life without financial stress, leading to higher overall wellbeing [4].

In contrast, countries like Italy, Spain, and Portugal, which have more limited pension systems and social safety nets, may leave retirees vulnerable to financial instability [51]. With pension systems under strain and growing life expectancy, retirees in these southern European countries often face higher financial stress and may struggle with healthcare access, which in turn negatively impacts their mental and physical wellbeing [32]. Similarly, in some eastern European countries (e.g., Poland or Hungary), retirees often experience a widening income gap, with many relying on modest state pensions, which can contribute to poorer mental health outcomes due to financial insecurity [51].

## 5. Conclusions and Future Research Directions

The limited number of studies in each category relating to aging and retirement is another noteworthy aspect of the results. These studies are becoming increasingly essential, as the findings highlight an increase in life expectancy, the aging of the population in Europe and, consequently, the extension of the time that individuals remain in the condition of retirement. Another aspect to be highlighted is the scarcity of articles addressing more psychological aspects of retirement and the preponderance of studies involving other issues related to the topic, such as physical health and economic issues.

This review hopes to have contributed with important information that could stimulate other studies on retirement and wellbeing promotion, especially works that address less explored themes, such as aging and retirement. A current description of research on retirement and wellbeing was presented and the phenomenon under study was situated with a certain scope, thus justifying the need for new studies that invest in the search for understanding this situation in Europe.

By highlighting factors that promote wellbeing in the retirement phase, the present study shows some indicators for the elaboration of intervention programs. At the level of primary prevention, in the phase before the reform, this regards indicators concerned with working on monitoring preparation for retirement. Additionally, the present study shows indicators for secondary prevention, specifically for those who are in the transition phase, and tertiary prevention for people who are already in the retirement process. In this, it seems to be important to reconcile measures of economic support and restructure more flexible working conditions, with advancing age and the approach of the transition to retirement negotiated with the workers. Moreover, future studies should create personalized program designs for each mentioned prevention phase, with an integral perspective, which respond to the needs highlighted in the present study, based on an integrative perspective of a biopsychosocial model of retirement, e.g., [52].

## Figures and Tables

**Table 1:** Risk of bias assessment.

NIH Quality Assessment Criteria	1	2	3	4	5	6	7	8	9	10	11	12	13	14	Quality Rating (Good, Fair, Poor)
Abramowska-Kmon, Łątkowski, and Rynko (2023) [14]	Yes	Yes	Yes	Yes	NR	N/A	N/A	N/A	Yes	N/A	Yes	N/A	N/A	NR	Fair
Adena, Hamermesh, Myck, and Oczkowska (2023) [15]	Yes	Yes	Yes	Yes	NR	N/A	N/A	N/A	Yes	N/A	Yes	N/A	N/A	Yes	Good
Arpino and Gómez-Léon (2020) [16]	Yes	Yes	Yes	Yes	NR	Yes	Yes	No	Yes	No	Yes	Yes	Yes	Yes	Good
Bađun and Smolic (2018) * [17]	Yes	Yes	Yes	Yes	NR	N/A	N/A	N/A	Yes	N/A	Yes	N/A	N/A	NR	Fair
Bauer, Brandt, and Kneip (2022) [18]	Yes	Yes	Yes	Yes	NR	N/A	N/A	N/A	Yes	N/A	Yes	N/A	N/A	Yes	Good
Cantisano, Depolo, León, and Domínguez (2009) * [19]	Yes	Yes	Yes	Yes	NR	N/A	N/A	N/A	Yes	N/A	Yes	N/A	N/A	Yes	Good
Cardoso and Brandão (2022) * [20]	Yes	Yes	Yes	Yes	NR	N/A	N/A	N/A	Yes	N/A	Yes	N/A	N/A	Yes	Good
Cheval et al. (2023) [21]	Yes	Yes	Yes	Yes	NR	N/A	N/A	N/A	Yes	N/A	Yes	N/A	N/A	Yes	Good
Danielsbacka, Tanskanen, Coall, and Jokela (2019) [22]	Yes	Yes	Yes	Yes	NR	N/A	N/A	N/A	Yes	N/A	Yes	N/A	N/A	NR	Fair
Dingemans and Henkens (2019) * [23]	Yes	Yes	Yes	Yes	NR	Yes	Yes	Yes	Yes	Yes	Yes	Yes	Yes	Yes	Good
Djundeva, Dykstra, and Fokkema (2019) [24]	Yes	Yes	Yes	Yes	NR	N/A	N/A	N/A	Yes	N/A	Yes	N/A	N/A	NR	Fair
Hank and Vagner (2013) [25]	Yes	Yes	Yes	Yes	NR	N/A	N/A	N/A	Yes	N/A	Yes	N/A	N/A	NR	Fair
Horner (2014) * [26]	Yes	Yes	Yes	Yes	NR	N/A	N/A	N/A	Yes	N/A	Yes	N/A	N/A	Yes	Good
Litwin and Levinsky (2023) [27]	Yes	Yes	Yes	Yes	NR	N/A	N/A	N/A	Yes	N/A	Yes	N/A	N/A	NR	Fair
Moreno-Agostino et al. (2020) * [28]	Yes	Yes	Yes	Yes	NR	N/A	N/A	N/A	Yes	N/A	Yes	N/A	N/A	Yes	Good
Okely, Cooper, and Gale (2016) [29]	Yes	Yes	Yes	Yes	NR	N/A	N/A	N/A	Yes	N/A	Yes	N/A	N/A	Yes	Good
Okely, Shaheen, Weiss, and Gale (2017) [30]	Yes	Yes	Yes	Yes	NR	N/A	N/A	N/A	Yes	N/A	Yes	N/A	N/A	Yes	Good
Okely, Weiss, and Gale (2018) [31]	Yes	Yes	Yes	Yes	NR	N/A	N/A	N/A	Yes	N/A	Yes	N/A	N/A	Yes	Good
Olivera and Ponomarenko (2017) [32]	Yes	Yes	Yes	Yes	NR	N/A	N/A	N/A	Yes	N/A	Yes	N/A	N/A	NR	Fair
Palomäki (2019) * [33]	Yes	Yes	Yes	Yes	NR	N/A	N/A	N/A	Yes	N/A	Yes	N/A	N/A	NR	Fair
Ploubidis and Grundy (2009) [34]	Yes	Yes	Yes	Yes	NR	N/A	N/A	N/A	Yes	N/A	Yes	N/A	N/A	Yes	Good
Ponomarenko (2016) * [35]	Yes	Yes	Yes	Yes	NR	N/A	N/A	N/A	Yes	N/A	Yes	N/A	N/A	Yes	Good
Ponomarenko, Leist, and Chauvel (2019) * [36]	Yes	Yes	Yes	Yes	NR	N/A	N/A	N/A	Yes	N/A	Yes	N/A	N/A	Yes	Good
Rojo-Perez et al. (2022) [37]	Yes	Yes	Yes	Yes	NR	N/A	N/A	N/A	Yes	N/A	Yes	N/A	N/A	Yes	Good
Siegrist, Wahrendorf, Knesebeck, Jurges, and Borsch-Supan (2006) [38]	Yes	Yes	Yes	Yes	NR	N/A	N/A	N/A	Yes	N/A	Yes	N/A	N/A	Yes	Good
Sohier (2019) * [39]	Yes	Yes	Yes	Yes	NR	N/A	N/A	N/A	Yes	N/A	Yes	N/A	N/A	Yes	Good
Sohier, van Ootegem, and Verhofstadt (2021) * [40]	Yes	Yes	Yes	Yes	NR	N/A	N/A	N/A	Yes	N/A	Yes	N/A	N/A	Yes	Good
Sohier, Defoor, Van Ootegem, and Verhofstadt (2022) [41]	Yes	Yes	Yes	Yes	NR	N/A	N/A	N/A	Yes	N/A	Yes	N/A	N/A	Yes	Good
Tambellini (2023) * [42]	Yes	Yes	Yes	Yes	NR	N/A	N/A	N/A	Yes	N/A	Yes	N/A	N/A	Yes	Good
Wahrendorf and Siegrist (2010) [43]	Yes	Yes	Yes	Yes	NR	N/A	N/A	N/A	Yes	N/A	Yes	N/A	N/A	Yes	Good
Weziak-Bialowolska, Skiba, and Bialowolski (2024) [44]	Yes	Yes	Yes	Yes	Yes	Yes	Yes	Yes	Yes	Yes	Yes	Yes	Yes	Yes	Good
Xia, van Wijngaarden, Huijsman, and Buljac-Samardžić (2024) [45]	Yes	Yes	Yes	Yes	NR	Yes	Yes	Yes	Yes	Yes	Yes	Yes	Yes	Yes	Good

Note. N/A, not applicable; NR, not reported. * Studies included in the meta-analysis.

**Table 2:** Characteristics of the included studies.

Publication Identification	European Countries	Type of Design	Age Group	Sample Size	Variables Involved
Abramowska-Kmon, Łątkowski, and Rynko (2023) [14]	Austria, Germany, Sweden, Spain, Italy, France, Denmark, Greece, Switzerland, Belgium, Czechia, Poland, Luxembourg, Portugal, Slovenia, Estonia, and Croatia.	Cross-sectional	50 years and older	30,179 participants (53.8% women)	Receiving informal care, subjective wellbeing
Adena, Hamermesh, Myck, and Oczkowska (2023) [15]	Austria, Belgium, Croatia, Czechia, Denmark, Estonia, France, Germany, Greece, Israel, Italy, Luxembourg, the Netherlands, Poland, Portugal, Slovenia, Spain, Sweden, and Switzerland	Cross-sectional	50 years and older	3056 participants (10.3% widowed)	Widowed and wellbeing, such as mental health and life satisfaction
Arpino and Gómez-Léon (2020) [16]	Austria, Belgium, Denmark, France, Germany, Greece, Israel, Italy, the Netherlands, Spain, Sweden, Switzerland	Retrospective cohort	Aged between 50 and 84 years old	11,796 participants (57.5% women)	Grandchild care, depressive symptoms, and wellbeing
Bađun and Smolić (2018) * [17]	Croatia	Cross-sectional	50 years and older	432 participants (40.4% women)	Early retirement wellbeing, health status
Bauer, Brandt, and Kneip (2022) [18]	Austria, Belgium, Croatia, Czech Republic, Denmark, Estonia, France, Germany, Greece, Hungary, Ireland, Italy, Luxembourg, the Netherlands, Poland, Portugal, Slovenia, Spain, Sweden, and Switzerland	Cross-sectional	50 to 85 years old	59,864 participants (55.9% women)	Parenthood and wellbeing
Cantisano, Depolo, León, and Domínguez (2009) * [19]	Austria, Denmark, France, Germany, Greece, Israel, Italy, Netherlands, Spain, Sweden, Switzerland, and Switzerland	Cross-sectional	50 years and older (mean age = 63 years old)	650 (47% women)	Bridge employment activity, job satisfaction, life satisfaction, and quality of life in retirement
Cardoso and Brandão (2022) * [20]	Austria, Belgium, Bulgaria, Croatia, Cyprus, Czech Republic, Denmark, Estonia, Finland, France, Germany, Greece, Hungary, Israel, Italy, Latvia, Lithuania, Luxembourg, Malta, The Netherlands, Poland, Portugal, Romania, Slovakia, Slovenia, Spain, Sweden, and Switzerland	Cross-sectional	Aged between 51 and 64 years old	10,794 participants (60% women)	Type 2 diabetes, retirement, wellbeing
Cheval et al. (2023) [21]	Austria, Belgium, Bulgaria, Croatia, Cyprus, Czech Republic, Denmark, Estonia, Finland, France, Germany, Greece, Hungary, Ireland, Israel, Italy, Latvia, Lithuania, Luxembourg, Malta, the Netherlands, Poland, Portugal, Romania, Slovakia, Slovenia, Spain, Sweden, and Switzerland—SHARE wave 1 to wave 8 (2004 to 2019)	Retrospective cohort	50 years and older	54,818 participants (55% women)	Educational attainment, quality of life wellbeing, physical activity, and depression symptoms
Danielsbacka, Tanskanen, Coall, and Jokela (2019) [22]	Austria, Belgium, Czechia, Denmark, France, Germany, Italy, the Netherlands, Spain, Sweden, and Switzerland	Retrospective cohort	Aged between 50 and 89 years old	41,713 participants (60.1% women)	Grandparental childcare and wellbeing, including difficulties with activities of daily living, depressive symptoms, life satisfaction, and meaning of life
Dingemans and Henkens (2019) * [23]	Austria, Belgium, Czechia, Denmark, Estonia, France, Germany, Hungary, Italy, the Netherlands, Poland, Portugal, Slovenia, Spain, Sweden, and Switzerland	Retrospective cohort	Aged between 60 and 75 years old	54,156 participants (53% women)	Working after retirement, wellbeing, and pension income
Djundeva, Dykstra, and Fokkema (2019) [24]	Austria, Belgium, Czechia, Denmark, Estonia, France, Germany, Hungary, Italy, the Netherlands, Poland, Portugal, Slovenia, Spain, Sweden, and Switzerland.	Cross-sectional	50 years and older	9904 participants	Social networks and subjective wellbeing, such as life satisfaction, satisfaction with social network, and depression
Hank and Vagner (2013) [25]	Austria, Belgium, Denmark, France, Greece, Germany, Italy, the Netherlands, Spain, Sweden, and Switzerland	Cross-sectional	65 years old and over	More than 9000 participants (54.2% women)	Parenthood, marital status, and wellbeing in later life
Horner (2014) * [26]	Austria, Belgium, Czechia, Denmark, France, Germany, Greece, Ireland, Italy, the Netherlands, Poland, Spain, Sweden, and Switzerland.	Cross-sectional	Aged between 50 and 70 years old	18,345 participants (only men)	Subjective wellbeing and retirement
Litwin and Levinsky (2023) [27]	Austria, Belgium, Bulgaria, Croatia, Cyprus, Czechia, Denmark, Estonia, Finland, France, Germany, Greece, Hungary, Israel, Italy, Latvia, Lithuania, Luxembourg, Malta, Poland, Slovakia, Slovenia, Spain, Sweden, and Switzerland	Cross-sectional	Aged 50 years and over	35,145 participants (58% women)	Perceived quality of life, wellbeing, depressive symptoms
Moreno-Agostino et al. (2020) * [28]	Finland, Poland, and Spain	Cross-sectional	Aged 50 years and over	2222 participants (46.95% women)	Experiential wellbeing, retirement
Okely, Cooper, and Gale (2016) [29]	Denmark, Sweden, Austria, France, Germany, Switzerland, Belgium, the Netherlands, Spain, Italy, and Greece	Cross-sectional	Aged 50 years and over	10,530 participants (51.66% women)	Wellbeing and arthritis incidence
Okely, Shaheen, Weiss, and Gale (2017) [30]	Denmark, Sweden, Austria, France, Germany, Switzerland, Belgium, the Netherlands, Spain, Italy, and Greece	Cross-sectional	Aged 50 years and over	12,246 participants (55.1% females)	Wellbeing and chronic lung disease incidence, retirement
Okely, Weiss, and Gale (2018) [31]	Austria, Belgium, Denmark, France, Germany, Greece, Israel, Italy, the Netherlands, Sweden, Switzerland, and Spain	Cross-sectional	Aged 50 years and over	13,596 participants (54.5% females)	Wellbeing, self-rated health
Olivera and Ponomarenko (2017) [32]	Austria, Germany, Belgium, Czechia, Denmark, Estonia, France, Greece, Hungary, Ireland, Italy, the Netherlands, Poland, Portugal, Slovenia, Spain, Sweden, and Switzerland	Cross-sectional	Aged between 50 and 75 years old	15,389 participants (50.5% women)	Pension insecurity and wellbeing
Palomäki (2019) * [33]	Austria, Belgium, Bulgaria, Croatia, Cyprus, the Czechia, Denmark, Estonia, Greece, Spain, Finland, France, Hungary, Ireland, Iceland, Italy, Lithuania, Luxembourg, Latvia, Malta, the Netherlands, Norway, Poland, Portugal, Romania, Sweden, Slovenia, Slovakia, and the United Kingdom	Cross-sectional	Aged 55 years and over	26,680 participants (58% women)	Subjective economic wellbeing, retirement
Ploubidis and Grundy (2009) [34]	Denmark, Sweden, Austria, France, Germany, the Netherlands, Spain, Italy, and Greece	Cross-sectional	50 years and over	13,498 participants (54.2% women)	Depression and wellbeing
Ponomarenko (2016) * [35]	Austria, Germany, Sweden, the Netherlands, Spain, Italy, France, Denmark, Greece, Switzerland, Belgium, Czech Republic, and Poland	Retrospective cohort	Aged 65 years and over	8098 participants (48.3% women)	Cumulative disadvantages of non-employment, non-standard work, retirement, and wellbeing
Ponomarenko, Leist, and Chauvel (2019) * [36]	Austria, Germany, Sweden, the Netherlands, Spain, Italy, France, Denmark, Switzerland, Belgium, Czechia, and Poland	Cross-sectional	Aged between 50 and 70 years old	2163 participants	Wellbeing, transition to retirement, employed, unemployed or economically inactive
Rojo-Perez et al. (2022) [37]	Spain	Cross-sectional	Aged 50 years and over	5566 participants (53.8% women)	Health, lifelong learning, quality of life, wellbeing
Siegrist, Wahrendorf, Knesebeck, Jurges, and Borsch-Supan (2007) [38]	Austria, Germany, Sweden, the Netherlands, Spain, Italy, France, Denmark, Greece, and Switzerland	Cross-sectional	65 years old or less	6836 participants (48.5% women)	Quality of work, wellbeing, and intended early retirement of older employees
Sohier (2019) * [39]	Denmark, Sweden, Belgium, Austria, France, Germany, the Netherlands, Spain, and Switzerland	Cross-sectional	Aged between 50 and 70 years old	16,667 participants	Working at older age, wellbeing
Sohier, van Ootegem, and Verhofstadt (2021) * [40]	Austria, Belgium, France, Germany, Spain, and Switzerland	Cross-sectional	Aged between 50 and 70 years old	38,344 participants	Life satisfaction, agency-freedom, wellbeing
Sohier, Defloor, van Ootegem, and Verhofstadt (2022) [41]	Austria, Belgium, Denmark, France, Germany, the Netherlands, Spain, Sweden, and Switzerland	Cross-sectional	Aged 50 and over	8410 participants	Personal facts, willingness to retire, wellbeing
Tambellini (2023) * [42]	Austria, Belgium, Denmark, France, Germany, Italy, the Netherlands, Poland, Spain, Sweden, and Switzerland	Cross-sectional	Aged 50 and over	2877 participants (100% women)	Transition to retirement and wellbeing
Wahrendorf and Siegrist (2010) [43]	Sweden, Denmark, the Netherlands, Germany, Belgium, France, Switzerland, Austria, Italy, Spain, and Greece	Cross-sectional	50 and over	10,309 participants (53.5% women)	Productive activities and wellbeing
Weziak-Bialowolska, Skiba, and Bialowolski (2024) [44]	Austria, Belgium, Czechia, Denmark, Estonia, France, Germany, Hungary, Italy, Poland, Slovenia, Spain, Sweden, Switzerland, and the Netherlands	Prospective cohort	50 and over	19,821 participants (59.29% women)	Voluntary and/or charity activities and cognitive impairment, daily life functioning, physical health, wellbeing
Xia, van Wijngaarden, Huijsman, and Buljac-Samardžić (2024) [45]	Austria, Belgium, Czechia, Denmark, France, Germany, Greece, Italy, Luxembourg, the Netherlands, Spain, Sweden, and Switzerland	Retrospective cohort	60 and over	4650 participants (65.16% women)	Support balance and subjective wellbeing, including depression, life satisfaction, and quality of life

* Studies included in the meta-analysis.

## Data Availability

The data used in this systematic review and meta-analysis were extracted from publicly available published studies. All data sources are properly referenced in the manuscript. Additional data can be obtained from the corresponding author upon request.

## References

[1] Eurostat (2019). Ageing Europe—Looking at the Lives of Older People in the EU.

[2] Pilehvari A., You W., Lin X. (2023). Retirement’s impact on health: What role does social network play?. Eur. J. Ageing.

[3] The Lancet Regional Health-Europe (2023). Securing the future of Europe’s ageing population by 2050. Lancet Reg. Health Eur..

[4] Kubicek B., Korunka C., Raymo J.M., Hoonakker P. (2011). Psychological well-being in retirement: The effects of personal and gendered contextual resources. J. Occup. Health Psychol..

[5] Henkens K., van Dalen H.P., Ekerdt D.J., Hershey D.A., Hyde M., Radl J., van Solinge H., Wang M., Zacher H. (2018). What We Need to Know About Retirement: Pressing Issues for the Coming Decade. Gerontologist.

[6] Wang M. (2007). Profiling retirees in the retirement transition and adjustment process: Examining the longitudinal change patterns of retirees’ psychological well-being. J. Appl. Psychol..

[7] Smeaton D., Barnes H., Vegeris S. (2017). Does Retirement Offer a “Window of Opportunity” for Lifestyle Change? Views From English Workers on the Cusp of Retirement. J. Aging Health.

[8] Wang M., Shultz K.S. (2010). Employee retirement: A review and recommendations for future investigation. J. Manag..

[9] Shultz K.S., Wang M. (2011). Psychological perspectives on the changing nature of retirement. Am. Psychol..

[10] Larsen M. (2008). Does quality of work life affect men and women’s retirement planning differently?. Appl. Res. Qual. Life.

[11] Moher D., Liberati A., Tetzlaff J., Altman D.G., PRISMA Group (2009). Preferred reporting items for systematic reviews and meta-analyses: The PRISMA statement. BMJ.

[12] National Institute of Health (2024). Quality Assessment Tool for Observational Cohort and Cross-Sectional Studies. https://www.nhlbi.nih.gov/health-topics/study-quality-assessment-tools.

[13] Weziak-Bialowolska D., Bialowolski P., Niemiec R.M. (2023). Character strengths and health-related quality of life in a large international sample. J. Res. Personal..

[14] Abramowska-Kmon A., Łątkowski W., Rynko M. (2023). Informal care and subjective well-being among older adults in selected European countries. Ageing Int..

[15] Adena M., Hamermesh D., Myck M., Oczkowska M. (2023). Home alone: Widows’ well-being and time. J. Happiness Stud..

[16] Arpino B., Gómez-Léon M. (2020). Consequences on depression of combining grandparental childcare with other caregiving roles. Aging Ment. Health.

[17] Bađun M., Smolić Š. (2018). Predictors of early retirement intentions in Croatia. Društvena Istraživanja.

[18] Bauer G., Brandt M., Kneip T. (2023). The role of parenthood for life satisfaction of older women and men in Europe. J. Happiness Stud..

[19] Cantisano G., Depolo M., León J., Domínguez J. (2009). Empleo puente y bienestar personal de los jubilados. Un modelo de ecuaciones estructurales con una muestra europea probabilística [Bridge employment and personal well-being of retirees. A structural equation model with a probabilistic European sample]. Psicothema.

[20] Cardoso M.F., Brandão M.P. (2022). Having type 2 diabetes does not imply retirement before age 65 in Europe. J. Popul. Ageing.

[21] Cheval B., Maltagliati S., Saoudi I., Fessler L., Farajzadeh A., Sieber S., Cullati S., Boisgontier M.P. (2023). Physical activity mediates the effect of education on mental health trajectories in older age. J. Affect. Disord..

[22] Danielsbacka M., Tanskanen A., Coall D., Jokela M. (2019). Grandparental childcare, health and well-being in Europe: A within individual investigation of longitudinal data. Soc. Sci. Med..

[23] Dingemans E., Henkens K. (2019). Working after retirement and life satisfaction: Cross-national comparative research in Europe. Res. Aging.

[24] Djundeva M., Dykstra P.A., Fokkema T. (2019). Is living alone “aging alone”? Solitary living, network types, and well-being. J. Gerontol. B Psychol. Sci. Soc. Sci..

[25] Hank K., Vagner M. (2013). Parenthood, marital status, and well-being in later life: Evidence from SHARE. Soc. Indic. Res..

[26] Horner E. (2014). Subjective well-being and retirement: Analysis and policy recommendations. J. Happiness Stud..

[27] Litwin H., Levinsky M. (2023). The interplay of personality traits and social network characteristics in the subjective well-being of older adults. Res. Aging.

[28] Moreno-Agostino D., Stone A., Schneider S., Koskinen S., Leonardi M., Naidoo N., Tobiasz-Adamczyk B., Haro J.M., Miret M., Kowal P. (2020). Are retired people higher in experiential wellbeing than working older adults? A time use approach. Emotion.

[29] Okely J., Cooper C., Gale C. (2016). Well-being and arthritis incidence: The survey of health, ageing and retirement in Europe. Ann. Behav. Med..

[30] Okely J., Shaheen S., Weiss A., Gale C. (2017). Well-being and chronic lung disease incidence: The survey of health, ageing and retirement in Europe. PLoS ONE.

[31] Okely J., Weiss A., Gale C. (2018). The interaction between individualism and well-being in predicting mortality: Survey of health ageing and retirement in Europe. J. Behav. Med..

[32] Olivera J., Ponomarenko V. (2017). Pension insecurity and well-being in Europe. J. Soc. Policy.

[33] Palomäki L. (2019). Does it matter how you retire? Old-age retirement routes and subjective economic well-being. Soc. Indic. Res..

[34] Ploubidis G.B., Grundy E. (2009). Later-life mental health in Europe: A country-level comparison. J. Gerontol. B Psychol. Sci. Soc. Sci..

[35] Ponomarenko V. (2016). Cumulative disadvantages of non-employment and non-standard work for career patterns and subjective well-being in retirement. Adv. Life Course Res..

[36] Ponomarenko V., Leist A.K., Chauvel L. (2019). Increases in well-being in the transition to retirement for the unemployed: Catching up with formerly employed persons. Ageing Soc..

[37] Rojo-Pérez F., Rodríguez-Rodríguez V., Molina-Martínez M.A., Fernandez-Mayoralas G., Sanchez-Gonzalez D., Rojo-Abuin J.M., Ayala A., Rodriguez-Blazquez C., Calderon-Larrañaga A., Ribeiro O. (2022). Active ageing profiles among older adults in Spain: A multivariate analysis based on SHARE study. PLoS ONE.

[38] Siegrist J., Wahrendorf M., Knesebeck O., Jurges H., Borsch-Supan A. (2007). Quality of work, well-being, and intended early retirement of older employees—Baseline results from the SHARE study. Eur. J. Public Health.

[39] Sohier L. (2019). Do involuntary longer working careers reduce well-being?. Appl. Res. Qual. Life.

[40] Sohier L., van Ootegem L., Verhofstadt E. (2021). Well-being during the transition from work to retirement. J. Happiness Stud..

[41] Sohier L., Defloor B., Van Ootegem L., Verhofstadt E. (2022). Determinants of the willingness to retire of older workers in Europe. Soc. Indic. Res..

[42] Tambellini E. (2023). Exploring the relationship between working history, retirement transition and women’s life satisfaction. Ageing Soc..

[43] Wahrendorf M., Siegrist J. (2010). Are changes in productive activities of older people associated with changes in their well-being? Results of a longitudinal European study. Eur. J. Ageing.

[44] Weziak-Białowolska D., Skiba R., Białowolski P. (2024). Longitudinal reciprocal associations between volunteering, health and well-being: Evidence for middle-aged and older adults in Europe. Eur. J. Public Health.

[45] Xia W., van Wijngaarden J.D.H., Huijsman R., Buljac-Samardžić M. (2024). The Effect of Long-Term (Im)balance of Giving Versus Receiving Support With Nonrelatives on Subjective Well-Being Among Home-Dwelling Older People. J. Gerontol. B Psychol. Sci. Soc. Sci..

[46] Feldman D.C. (1994). The decision to retire early: A review and conceptualization. Acad. Manag. Rev..

[47] Merkel S., Ruokolainen M., Holman D. (2019). Challenges and practices in promoting (ageing) employees’ working careers in the health care sector: Case studies from Germany, Finland, and the UK. BMC Health Serv. Res..

[48] Lahdenperä M., Virtanen M., Myllyntausta S., Pentti J., Vahtera J., Stenholm S. (2022). Psychological Distress During the Retirement Transition and the Role of Psychosocial Working Conditions and Social Living Environment. J. Gerontol. B Psychol. Sci. Soc. Sci..

[49] Maslach C., Leiter M.P. (2016). Understanding the burnout experience: Recent research and its implications for psychiatry. World Psychiatry.

[50] Hartanto A., Sim L., Lee D., Majeed N.M., Yong J.C. (2024). Cultural contexts differentially shape parents’ loneliness and wellbeing during the empty nest period. Commun. Psychol..

[51] Bambra C., Eikemo T.A. (2009). Welfare state regimes, unemployment and health: A comparative study of the relationship between unemployment and self-reported health in 23 European countries. J. Epidemiol. Community Health.

[52] White M.P., Hartig T., Martin L., Pahl S., van den Berg A.E., Wells N.M., Costongs C., Dzhambov A.M., Elliott L.R., Godfrey A. (2023). Nature-based biopsychosocial resilience: An integrative theoretical framework for research on nature and health. Environ. Int..

